# Alar Asymmetry in Patients with Unilateral Cleft Lip: Implications for Secondary Rhinoplasty

**DOI:** 10.1177/10556656231168769

**Published:** 2023-04-04

**Authors:** Lucas M. Harrison, Laura Kenyon, Denzil P. Mathew, Christopher A. Derderian, Rami R. Hallac

**Affiliations:** 1 Department of Plastic Surgery, University of Texas Southwestern Medical Center, Dallas, TX, USA; 2 Analytical Imaging and Modeling Center, Children's Medical Center, Dallas, TX, USA

**Keywords:** cleft lip, nasal morphology, rhinoplasty

## Abstract

**Objective:**

Alar asymmetry in unilateral cleft lip (UCL) nasal deformity is a well-recognized clinical feature. However, there is a lack of comprehensive quantitative analysis of this asymmetry. This study compares the shape, volume, and axis rotation between the cleft and non-cleft ala in skeletally mature patients with UCL.

**Design:**

A retrospective comparative study utilizing three-dimensional rendered CT scans.

**Setting:**

Tertiary care pediatric institution.

**Patients, Participants:**

This study included 18 patients with UCL nasal deformity at skeletal maturity.

**Main Outcome Measure(s):**

Cleft and non-cleft side ala volume, surface area, and axis to the midsagittal plane.

**Results:**

The cleft-side ala was significantly lesser in volume by 27.3%, significantly lesser in surface area by 17.6%, and significantly greater in surface area to volume ratio by 14.6% than the non-cleft ala. The cleft-side ala was significantly greater by 43.1% horizontal axis to the midsagittal plane. In patients with primary rhinoplasty, the cleft-side ala had 28.0% less volume and 18.7% less surface area. In intermediate rhinoplasty, the cleft-side ala had 39.1% less volume and 23.5% less surface area than the non-cleft ala.

**Conclusions:**

Significant asymmetry exists between the cleft-side and non-cleft ala in patients with UCL. The cleft-side ala is significantly smaller in volume and surface area than the non-cleft ala. Additionally, the cleft-side ala demonstrates a significantly greater horizontal axis that contributes considerably to nasal asymmetry, supporting the need to restore a normal vertical axis to the clef-side ala.

## Introduction

The cleft-side nasal ala in the unilateral cleft lip (UCL) has a baseline deformity due to a discontinuous orbicularis oris, skeletal disruption, and asymmetry of the maxilla.^1^ The nasal asymmetry associated with UCL is a complex three-dimensional deformity resulting in significant aesthetic and functional problems. The nose's appearance is often the most stigmatizing feature for patients with UCL, with asymmetry of the nasal tip and nostrils. As patients grow, the asymmetry of the nose is observed to worsen due to the deformity of the nasal cartilage, scar tissue from previous surgeries, and persistent asymmetry at the pyriform aperture. Once skeletal maturity and normal occlusion are achieved, secondary rhinoplasty is frequently undertaken to address the asymmetry of the nose.^2,3^ Surgeons often employ cartilage grafting, scar modifications, and alar base narrowing. Secondary cleft rhinoplasty is much more complex than primary rhinoplasty due to the unfavorable soft tissue envelope and the significant asymmetry of the nose, including the cleft side ala.^4,5^

Prior studies have described UCL nasal asymmetry using standard nasolabial indices such as nostril width ratio, lateral lip height ratio, and columellar angle before and after secondary rhinoplasty.^6,7^ Although the diminutive size of the cleft-side ala, compared to the non-cleft ala, is a well-recognized feature of UCL, limited quantitative analysis of this asymmetry has been reported. This study aims to quantitatively compare the shape, volume, and axis rotation of the cleft-side ala to the contralateral non-cleft ala in skeletally mature patients with UCL to understand how we may address alar asymmetry at the time of secondary cleft rhinoplasty.

## Methods

Following institutional review board approval #STU-042013-005 by the University of Texas Southwestern Medical Center and written consent, three-dimensional (3D) images were acquired using computed tomography (CT) head images from skeletally mature patients with UCL nasal deformity.^8^ Patients with previous secondary rhinoplasty or other congenital or acquired facial deformities were excluded. Eighteen patients with UCL met the inclusion criteria. The average age was 16.81  ±  1.18 years. A total of 10 were males, and 8 were females. The cleft lip was left-sided in 11 patients and right-sided in 7 patients.

**Figure 1.**
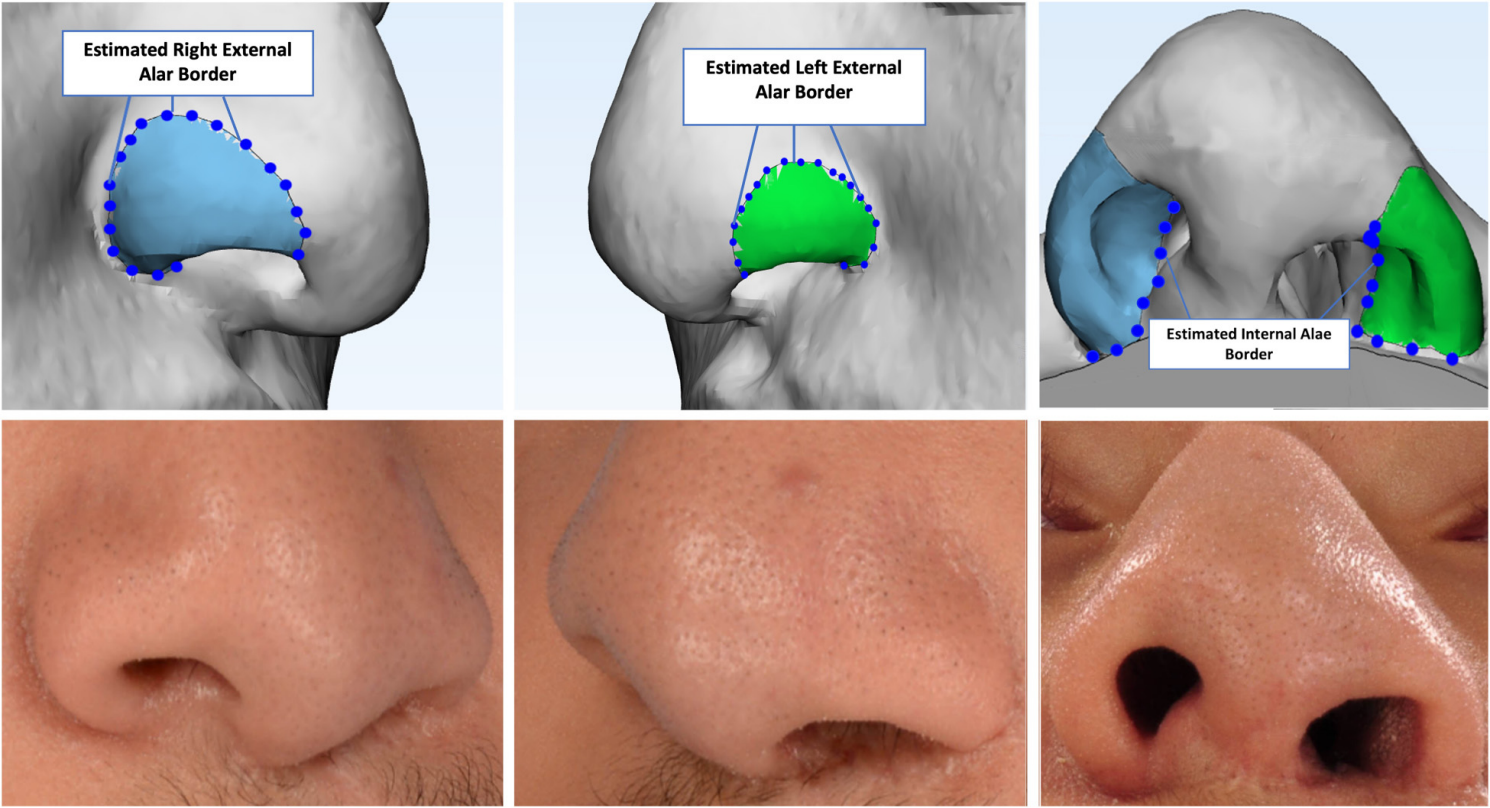
The soft tissue model correlated with the patient's clinical photo depicting segmented cleft-side ala (left column) and non-cleft ala (middle column). Patient's estimated external and internal alar borders (right column).

Three-dimensional (3D) rendered soft tissue models of CT head images were created using Materialise Mimics software (Materialise NV, Leuven, Belgium). The cleft-side and non-cleft ala were outlined and segmented based on reproducible anatomical landmarks. The external alar border was estimated using two-dimensional (2D) images of the patient and tissue thickness (Figure 1). The internal alar border was estimated based on the caudal edge of the lower lateral cartilage. The volume and surface area were calculated utilizing the imaging software. We performed intra-rater reliability testing by repeating the landmark placement and segmentation for five randomly chosen patients.

**Figure 2.**
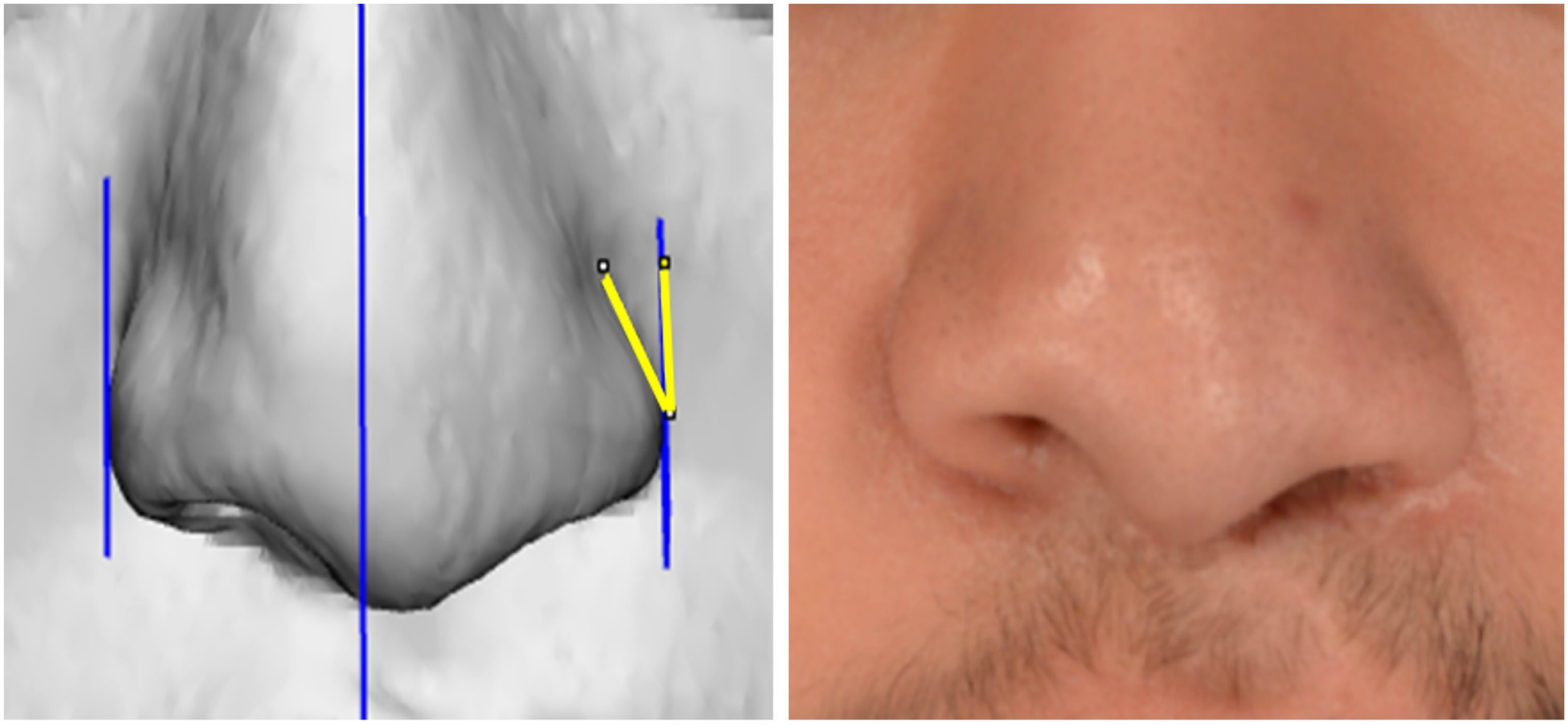
Two-dimensional rotation angle from the patient's lateral alar rim plane (parallel to the midsagittal plane) to the cleft ala.

Each CT scan was converted into a 3D bone model to determine the midline of the skull. The midsagittal and parallel planes were created at the lateral alar rim on the 3D model. After aligning the 3D model to the Frankfurt plane, the model was uploaded into ImageJ (U.S. National Institutes of Health, Bethesda, MD), and the 2D rotation angle from the lateral alar rim plane to the ala was calculated. (Figure 2). The angle calculated from the lateral alar rim to each ala represents alar orientation or axis of rotation.

The measurements for volume, surface area, and angles of the cleft and non-cleft sides were compared for each patient, and statistical analysis was performed. The data were subdivided into primary, intermediate, and no rhinoplasty to evaluate the effect of the timing of rhinoplasty. Additionally, the data were analyzed based on the operating surgeon. A Student's t-test with a significance level of α < 0.05 was used for all measurements and intra-rater reliability was tested using a linear regression model.

## Results

Our analysis revealed that the cleft-side ala has significantly less volume and surface area than the non-cleft ala. The mean volume of the cleft-side ala was significantly less by 27.0% than the non-cleft ala (p < 0.001). The surface area was also significantly less by 17.6% on the cleft side ala in comparison to the non-cleft ala (p < 0.001) (Table 1). The cleft-side ala surface area to volume ratio was significantly greater by 13.2% than the non-cleft ala ratio (p < 0.001). The intra-rater reliability test indicated a consistent method of obtaining volume and surface area with a correlation coefficient of 0.96 and 0.98. The cleft-side ala axis decreased vertically while increasing horizontally compared to the non-cleft ala. The cleft ala had a significantly greater axis of rotation (p < 0.001) to the midsagittal plane (36.0°  ±  6.9°) when compared to the non-cleft ala (25.2°  ±  6.6°), indicating a 30.0% increase.

**Table 1.** Cleft and non-Cleft Alar Volume, Surface Area, Surface Area to Volume Ratio, and Axis of Rotation.

	Cleft-side Ala	Non-cleft Ala	p Value
Volume	880.7 ± 331.7 mm^2^	1205.6 ± 370.4 mm^2^	<0.001*
Surface Area	634.0 ± 145.1 mm^2^	769.7 ± 147.8 mm^2^	<0.001*
SA:V Ratio	0.76	0.66	<0.001*
Axis	36.0 ± 6.9**°**	25.2 ± 6.6**°**	<0.001*

Surface Area to Volume (SA:V), Statistically Significant (*).

Of the 18 patients in our study, ten patients had primary rhinoplasty, two had intermediate rhinoplasty, and six did not have either (Table 2). We found that in patients with primary rhinoplasty, the cleft-side ala had 28.0% less volume and 18.7% less surface area than the non-cleft ala. In intermediate rhinoplasty, the cleft side had 39.1% less volume and 23.5% less surface area than the non-cleft ala. Although various surgical techniques were used for the primary cleft rhinoplasty, our data revealed similar asymmetries when comparing the cleft-side ala to the non-cleft ala (Table 3).

**Table 2.** Differences in Rhinoplasty Timing in Cleft and non-Cleft Alar Volume and Surface Area.

	Primary Rhinoplasty	Intermediate Rhinoplasty	No Rhinoplasty
Number of Patients	10	2	6
Non-cleft Volume	1357.8 mm^2^	1236.0 mm^2^	941.7 mm^2^
Cleft-side Volume	985.2 mm^2^	769.4 mm^2^	743.8 mm^2^
Percent Volume Difference	28.0%	39.1%	22.0%
Non-cleft SA	822.3 mm^2^	764.9 mm^2^	683.7 mm^2^
Cleft-side SA	668.3 mm^2^	585.7 mm^2^	592.9 mm^2^
Percent SA Difference	18.7%	23.5%	13.7%

Surface Area (SA).

**Table 3.** Differences in Cleft and non-Cleft Alar Volume Surface Area, and Axis by Surgeon.

	Volume	Surface Area	Axis
Surgeon 1			
Cleft Side Ala	986.1 mm^3^	661.9 mm^2^	35.4°
Non-cleft Side Ala	1356.7 mm^3^	815.1 mm^2^	25.8°
Percent Difference	28.2%	18.9%	42.6%
Surgeon 2			
Cleft Side Ala	946.2 mm^3^	692.4 mm^2^	41.1°
Non-cleft Side Ala	1271.8 mm^3^	822.6 mm^2^	31.9°
Percent Difference	25.7%	15.9%	29.7%
Surgeon 3			
Cleft Side Ala	1056.6 mm^3^	664.6 mm^2^	28.6°
Non-cleft Side Ala	1537.5 mm^3^	872.0 mm^2^	19.2°
Percent Difference	31.3%	23.8%	48.9%

## Discussion

The study found significant asymmetry between the cleft-side and non-cleft ala in patients with UCL, with the cleft-side ala being significantly smaller in volume and surface area and having a significantly greater horizontal axis. These findings support the need to restore a normal vertical axis to the cleft-side ala to improve the nose's symmetry in patients with UCL. The study also found that primary and intermediate rhinoplasty had a limited effect on the size and shape of the cleft-side ala, suggesting that more advanced surgical techniques may be needed to achieve a more symmetric nose.

Several factors cause the nasal asymmetry characteristic of unilateral cleft lip. Fisher and Mann described the lobule as having two arches – one cartilaginous and one made up of the soft tissue of the nostril margin.^9,10^ In this model, a wider cleft deformity leads to more significant posterolateral displacement of the cartilaginous ring. The increased traction on the nostril's soft tissue margin causes the ala recurvatum deformation.^9,10^ Tension at the interface of these two arches results in the formation of the vestibular web. Release of the lower lateral cartilage from the piriform aperture in conjunction with the soft tissue attachments of the alar base during cleft lip repair allows medial repositioning of the alar base into an asymmetric position non-cleft alar base. While these maneuvers allow repositioning of the ala, they also require the complete release of the pyriform ligament.^11,12^ The pyriform ligament is a critical support structure that provides stability to the ala through the attachment of the ala to the pyriform aperture. Loss of this support may contribute to the horizontal axis of the cleft-side ala observed in this study.

Similarly, the persistent skeletal asymmetry present after cleft lip repair frequently results in malposition of the lateral crus after cleft lip repair. The resulting tension on the lateral crus depresses the nostril rim, which also likely contributes to the more horizontal axis of the ala observed in the current study. Our study supports the use of surgical techniques, such as inserting a non-anatomic lateral crural strut graft or using an alar batten graft, to correct this abnormal horizontal axis and improve alar symmetry. However, it is important to note that the horizontal axis of the ala is just one aspect of alar asymmetry, and a comprehensive approach to cleft lip repair is necessary to address all aspects of the deformity.

We found a significant volume discrepancy between the cleft-side and non-cleft ala in patients with UCL. The cleft-side ala is significantly smaller and more transversely oriented, accentuating the vertical asymmetry between the cleft and non-cleft ala. The cleft-side ala had 27.0% less volume compared to the non-cleft ala. The surface area was 17.6% less than the non-cleft ala, and the surface area to volume ratio was 13.2% greater on the cleft side (p < 0.001). While a greater surface area to volume ratio could mean that the cleft side ala is thinner than the unaffected side, this is not typically observed clinically. Another likely explanation is that the surface area to volume ratio is related to the smaller size of the cleft ala.

Recently, Tse and colleagues presented the foundation approach to improving nasal asymmetry at the time of cleft lip repair.^13^ This approach addresses the lateral malposition of the non-cleft ala and nasal septum away from the midline toward the non-cleft side, resulting in a less radical medial repositioning of the cleft-side alar base. Primary cleft rhinoplasty is not performed in their approach. Early evidence suggests that the foundation approach provides stable symmetry in the nasal base five years after surgery.^14^ Their group has not evaluated the volume of cleft-side and non-cleft ala. However, the symmetry achieved with their delayed approach to primary rhinoplasty raises the question of whether adverse sequelae of some techniques for primary rhinoplasty may contribute to the significant alar asymmetry observed in our study.

Many techniques are used for primary cleft rhinoplasty, which are designed to correct nasal asymmetry. Maneuvers to approximate the domes are a common theme. However, additional maneuvers to reposition the lateral crus, suturing the lateral crus to the upper lateral cartilage, alar rim incisions and changes in the alar base or vestibular web anatomy have also been described.^15–17^ However, despite the variety of techniques used, analysis of the patients in our cohort by surgeon showed comparable asymmetry in the alar volume. It is worth noting that two patients with an intermediate rhinoplasty had the most considerable differences in volume. This suggests that the asymmetry of the ala contributed to the need for intermediate rhinoplasty, but this could not be determined. Overall, there was a comparable asymmetry in the alar volume of the entire cohort, regardless of whether primary rhinoplasty was performed.

This study found that there can be significant asymmetry in the ala at the time of secondary rhinoplasty, but it could not determine if this asymmetry results from the congenital deformity or primary cleft rhinoplasty. However, the alar volume discrepancy was similar among three surgeons who used different techniques for primary cleft rhinoplasty. These findings suggest that appreciation of the size differential in the ala should be considered during nasal analysis and factored into the surgical plan to achieve symmetry. The data from this study support the use of an alar base resection on the non-cleft side to address any residual alar asymmetry present after lateral crural strut graft or alar batten grafting. Further research is needed to understand how these surgical maneuvers affect the perception of nasal and alar symmetry.

## Conclusion

Significant asymmetry exists between the cleft and non-cleft ala in patients with UCL. The cleft-side ala is significantly smaller in volume and surface area than the non-cleft ala. The cleft-side ala also demonstrates a more significant horizontal axis than the non-cleft ala, and this variable showed the most significant difference in the symmetry between the cleft and non-cleft sides. This asymmetry did not appear to differ with varying techniques for primary cleft rhinoplasty. These findings support the use of surgical maneuvers to restore a normal vertical axis to the cleft-side ala and suggest that an alar base resection from the non-cleft side at the time of secondary cleft rhinoplasty may improve nasal symmetry.
